# Genome Sequence of *Bacillus safensis* Strain Ingolstadt Isolated from the Pectoralis Pouch of a Patient with Defibrillator-Related Surgery

**DOI:** 10.1128/genomeA.01031-17

**Published:** 2017-09-21

**Authors:** Flavia Dematheis, Markus H. Antwerpen, Gregor Grass, Mathias C. Walter, Stefan Borgmann

**Affiliations:** a Bundeswehr Institute of Microbiology, Munich, Germany; b Department of Infectious Diseases and Infection Control, Klinikum Ingolstadt, Ingolstadt, Germany

## Abstract

We report the draft genome sequence of clindamycin-resistant *Bacillus safensis* strain Ingolstadt isolated from a patient with bacterial colonization after heart surgery. The draft genome comprises 3.75 Mbp and harbors 3,793 predicted protein-encoding genes and a small plasmid.

## GENOME ANNOUNCEMENT

The *Bacillus pumilus* group comprises five species, *B. pumilus*, *B. altitudinis B. aerophilus*, *B. safensis*, and *B. stratosphericus* (1). This group contains soil bacteria, and some strains of *B. pumilus sensu stricto* cause severe infections. The wide range of reported cases includes cutaneous lesions, anthrax-like necrosis, catheter infections, and severe sepsis in neonatal infants (2–4). *Bacillus safensis* strains have been characterized as surfactant producers (5) and as petroleum degraders employing excreted lipolytic exoenzymes (6). Because of the bacterium’s resistance against disinfection with hydrogen peroxide, other strains have been isolated from clean surfaces of spacecraft and their production facilities (7). Here, we report the genome sequence of a bacillus initially identified as *B. pumilus* by use of matrix-assisted laser desorption ionization time of flight (MALDI-TOF). This isolate turned out to be a *B. safensis* strain (7) which had caused internal colonization of a patient treated at the general hospital in Ingolstadt, Germany.

*B. safensis* strain Ingolstadt was isolated from a smear withdrawn on 24 January 2017 from the pectoralis pouch of a defibrillator implanted in a 66-year-old man. The patient had received the defibrillator some weeks earlier. During a second surgery in 2017, the defibrillator was refitted with a new electrode located within the epicardium of the left ventricle. Antimicrobial therapy with cefazolin and rifampin prevented persistence of the bacteria and colonization of the new electrode. No lesions or any other tissue damage was visible, which differs from earlier reports of symptoms associated with infections with closely related *B. pumilus sensu stricto* in humans (2–4).

Whole-genome shotgun (WGS) sequencing of *B. safensis* strain Ingolstadt was performed with Illumina MiSeq sequencing technology (Illumina Inc.) using Nextera V3 2- × 300-bp chemistry. High-quality paired-end reads were assembled *de novo* using an in-house script based on SPAdes assembler (8) and Pilon (9) for genome assembly correction. A total of 3,754,517 bp were assembled in 17 contigs with an *N*_50_ value of 954,574 and 41.5% GC content.

A total of 3,793 coding sequences (CDSs) of the draft genome of *B. safensis* Ingolstadt were annotated using Prokka (10). RNAmmer software (11) was used to predict 5S, 16S, and 23S ribosomal RNAs. Eight copies of 5S, 16S, and 23S rRNA and 72 tRNA loci were identified. The closest genome relative to strain Ingolstadt in the NCBI database was *B. safensis* strain KCTC (12,796 bp) (GenBank accession number CP018197), with an average sequence identity of 96.6% (12).

*B. safensis* Ingolstadt harbors a small plasmid (7,241 bp, contig 15) that shares about 97% similarity with *B. pumilus* strain GR-8 plasmid pGR-8 (accession number CP009109). The plasmid harbors six CDSs but lacks any obvious virulence factors. Antimicrobial susceptibility testing of strain Ingolstadt revealed resistance against the lincosamide antibiotic clindamycin (MIC >4 mg/µl as determined by microdilution assays). No *cfr*-like genes (13), which are known to confer lincosamide resistance through ribosome methylation, were identified on the genome. Similarly, sequence analysis of the 23S rRNA, *rplD* (L4 r-protein), and *rplC* (L3 r-protein) genes of strain Ingolstadt did not show any single-nucleotide variant known to contribute to lincosamide resistance in other Gram-positive bacteria (14).

### Accession number(s).

This whole-genome shotgun project has been deposited at DDBJ/ENA/GenBank under the accession number NMQS00000000. The version described in this paper is version NMQS01000000.
